# Supplementary material for: The Phylogeny and Metabolic Potentials of a Lignocellulosic Material-Degrading Aliiglaciecola Bacterium Isolated from Intertidal Seawater in East China Sea
Source: Microorganisms. 2024 Jan 11;12(1):144. doi: 10.3390/microorganisms12010144 (PMC10821302; doi:10.3390/microorganisms12010144)

Supplementary Tables and Figures:

Supplementary Tables:

Table S1: The ANIb values and aligned percentage between strain LCG003 and other strains.

Table S2: The AAI values and aligned percentage between strain LCG003 and other strains.

Table S3: The DDH value and aligned percentage between strain LCG003 and other strains.

Table S4: The COG classification of strain LCG003 and other three *Aliiglaciecola* strains.

Table S5: The 12 genomic islands found in strain LCG003.

Table S6: The genes of transposases, integrases and/or recombinases predicted in strain LCG003.

Table S7: The glycoside hydrolase genes predicted in strain LCG003.

Table S8: The extracellular peptidases predicted in strain LCG003.

Table S9: The genes involved in degradation of 4-hydroxybenzoate and vanillate degradation.

Supplementary Figures:

Figure S1: The transmission electron micrograph of strain LCG003.

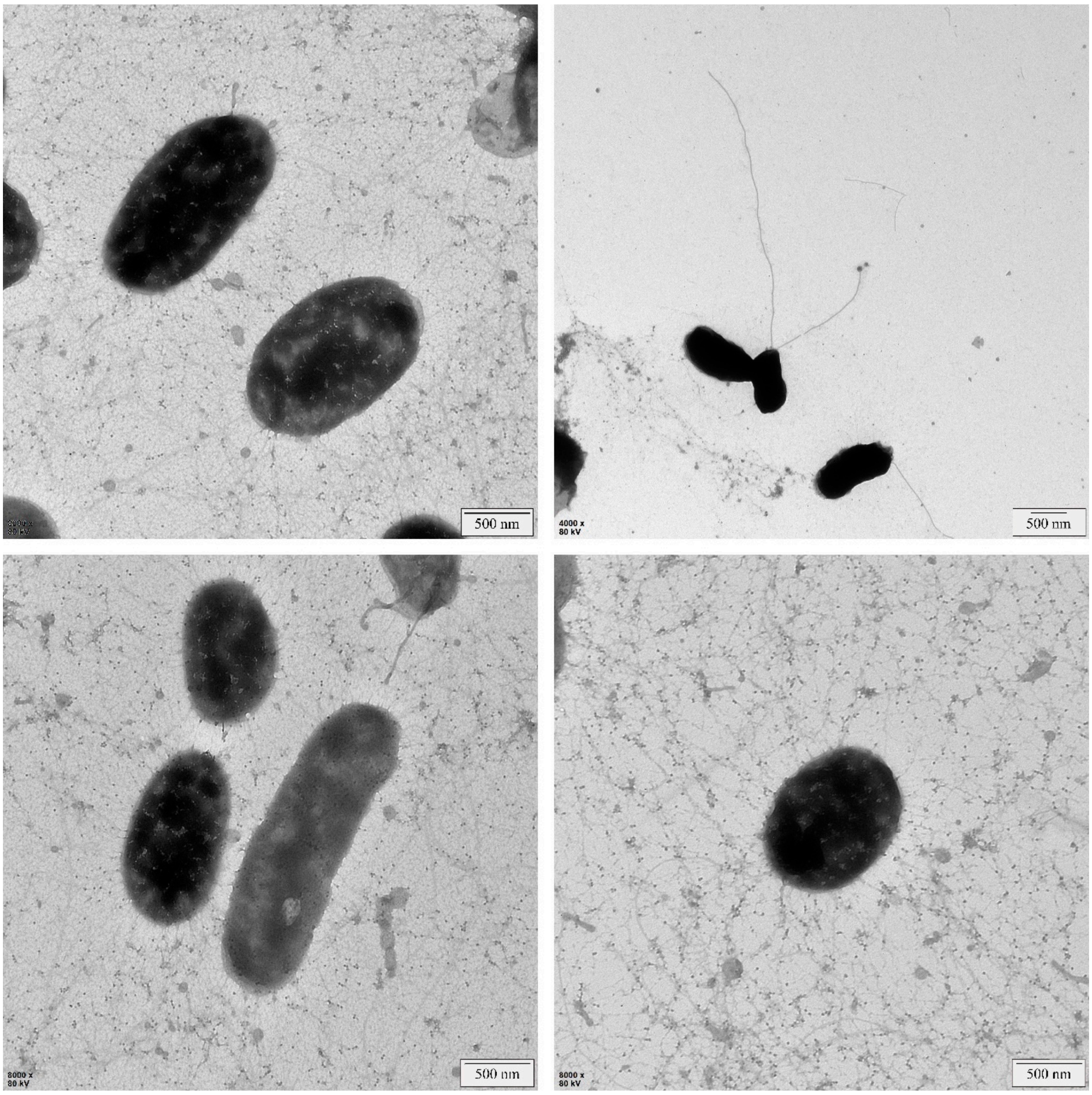

Figure S2: The growth curve of strain LCG003 under multiple temperatures.

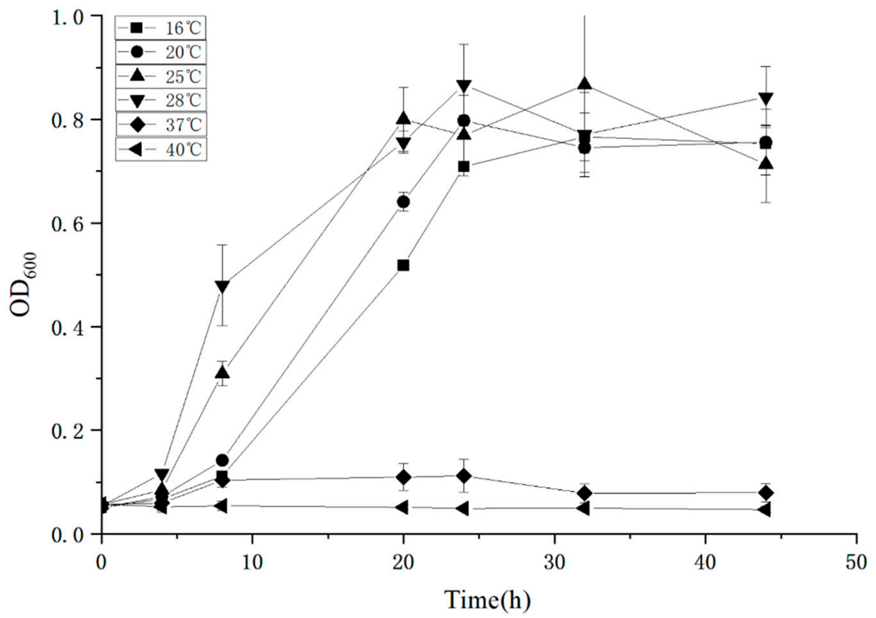

Figure S3: The lignin utilization of strain LCG003. A and B are experimental groups, and C and D are control groups, indicating the counts of live bacteria of strain LCG003 calculated using the SYTO 9/Pl live/dead bacteria double strain kit with ASW-lignin medium for 0 and 7 days, respectively. Error bar indicate each sample was with three independent biological replicates. Asterisks indicate t-test significant differences (NS, no significant; *, P value < 0.05; **, P value < 0.01; ***, P value < 0.001; ****, P value < 0.0001).

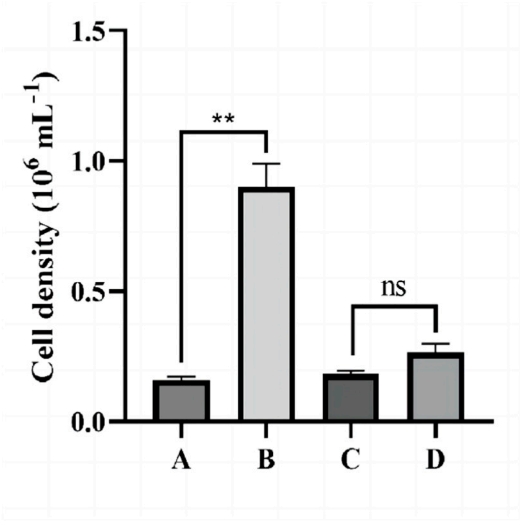

Supplement: Supplementary file 1 [file microorganisms-12-00144-s001.zip › Supplementary Tables and Figures Legends.pdf]
